# Chemopreventive Effect of β-Cryptoxanthin on Human Cervical Carcinoma (HeLa) Cells Is Modulated through Oxidative Stress-Induced Apoptosis

**DOI:** 10.3390/antiox9010028

**Published:** 2019-12-27

**Authors:** Enkhtaivan Gansukh, Arti Nile, Iyyakkannu Sivanesan, Kannan R. R. Rengasamy, Doo-Hwan Kim, Young-Soo Keum, Ramesh Kumar Saini

**Affiliations:** 1 Department of Bioresources and Food Science, Konkuk University, Seoul 143-701, Korea; enkhtaivan11@naver.com (E.G.); aartibmahajan@gmail.com (A.N.); isivanesan@gmail.com (I.S.); rengasamy@iceir.net (K.R.R.R.); kimdh@konkuk.ac.kr (D.-H.K.); 2 Institute of Natural Science and Agriculture, Konkuk University, Seoul 143-701, Korea; 3 Department of Crop Science, Konkuk University, Seoul 143-701, Korea; rational@konkuk.ac.kr

**Keywords:** carotenoids, DNA fragmentation, mitochondrial membrane potential, ROS-induced apoptosis, TUNEL assay

## Abstract

The present study was aimed to assess cellular and molecular events involved in the chemopreventive activities of β-cryptoxanthin derived from mandarin oranges (*Citrus unshiu* Marc.) on human cervical carcinoma (HeLa) cells. In vitro experiments established that β-cryptoxanthin significantly inhibited the proliferation of HeLa cells with the IC_50_ value of 4.5 and 3.7 µM after 24 and 48 h of treatments, respectively. β-cryptoxanthin-treated HeLa cells exhibited enhanced levels of oxidative stress correlated with significant downregulation of anti-apoptotic Bcl-2, and upregulation of pro-apoptotic Bax mRNA expression. Moreover, β-cryptoxanthin triggered nuclear condensation and disruption of the integrity of the mitochondrial membrane, upregulated caspase-3, -7, and -9 mRNA, and enhanced activation of caspase-3 proteins, resulting in nuclei DNA damage and apoptosis of HeLa cells. Remarkably, TUNEL assay carried out to detect nuclei DNA damage showed 52% TUNEL-positive cells after treatment with a physiological concentration of β-cryptoxanthin (1.0 μM), which validates its potential as an anticancer drug of natural origin.

## 1. Introduction

Dietary intake of fruits and vegetables that are rich in bioactive carotenoids and other antioxidant phytochemicals (e.g., polyphenols, terpenoids, and isothiocyanantes) is associated with lower risk of chronic conditions and diseases, such as age-related macular degeneration, cardiovascular diseases (CVD), diabetes, neurodegenerative disorders, and several types of cancer [1,2,3,4,5,6,7]. Among females, cervical cancer is the fourth most commonly diagnosed malignancy in women worldwide, after malignancy of breast, colorectal, and lungs [8]. The efforts to discover phytochemicals-based chemopreventive and chemotherapy drugs for cervical cancer and other types of cancer have been increasing in recent years [3]. In this regard, several antioxidant phytochemicals, including carotenoids, have shown promising results of pro-apoptotic, anti-proliferative, anti-metastatic, and anti-angiogenic effects on various cancer types [9]. Our recent results revealed that lutein isolated derived from marigold petals trigger ROS production, inhibit proliferation, and interact with mitochondrial B-cell lymphoma (Bcl)-2 family proteins to activate the caspase-3 mediated apoptosis of human cervical carcinoma HeLa cells [10]. Similarly, several xanthophylls, including 9-*Z*-neoxanthin, showed potent and selective cytotoxic effects against HeLa cells, with IC_50_ values of just 3.8 µM [11].

β-cryptoxanthin (other names: β,β-Caroten-3-ol) is a provitamin A carotenoid that widely occurs in paprika, squash, persimmons, oranges, papayas, and peaches (USDA Nutrient Database for Standard Reference, Release 28). Despite more than 40 carotenoids found in the diet, only six carotenoids, including three non-provitamin A (lycopene, lutein, and zeaxanthin) and three pro-vitamin A carotenoids (α-carotene, β-carotene, and β-cryptoxanthin) represent ≈ 95% of total blood carotenoids [12]. In plasma, lycopene is most abundantly found ((0.43–1.32) μM; recorded among Europeans), followed by lutein, β-carotene, and β-cryptoxanthin (0.11–0.52 μM) [13]. This fourth highest occurrence of β-cryptoxanthin in plasma is unique, since it occurs in a limited number of foods that are not dietary staples. Mandarin oranges, papaya, sweet potato leaf, watermelon, and related products are the key contributor of β-cryptoxanthin in the human diet [14].

To the best of our knowledge, no comprehensive studies were available on the effect of provitamin A carotenoids β-cryptoxanthin on cervical cancer. However, several epidemiological in vitro and in vivo studies have demonstrated the protective role of β-cryptoxanthin in several types of cancer, including lung [15,16,17], stomach [18], and human colon adenocarcinoma [19]. In mechanistic studies, β-cryptoxanthin triggered antitumor activities were largely modulated by significantly enhanced production and accumulation of reactive oxygen species (ROS) and reactive nitrogen species (RNS) [19], decrease in cell viability, cell cycle arrest by downregulations of cell cycle proteins cyclin D1 and cyclin E [20], increased activities of p21 cyclin-dependent kinase inhibitor and retinoic acid receptor (RAR)-β [18], activation of Bcl-2-associated death promoter (Bad) protein, suppressed cell migration, and caspase-triggered apoptosis [19].

Recognizing the above described cytotoxic properties of β-cryptoxanthin against several cancer types, we, therefore, hypothesized that β-cryptoxanthin display cytotoxicity against cervical cancer HeLa cells by modulating pathways involved in cellular proliferation. To test this hypothesis, cellular ROS levels, mitochondrial membrane integrity, the mRNA expression profile of anti-apoptotic Bcl-2 (B-cell lymphoma-2) and pro-apoptotic tumor protein p53 (p53), Bcl-2-associated X protein (Bax), and caspase-3, -7, and -9 proteins were studied in HeLa cells exposed to β-cryptoxanthin. The expression of active (cleaved) caspase-3 proteins were also studied. Additionally, the integrity of apoptotic HeLa cell nuclear DNA was investigated to determine the potential impact of β-cryptoxanthin treatment on programmed cell death.

## 2. Materials and Methods

### 2.1. Plant Material and Reagents

β-cryptoxanthin used in the experiments was isolated from the pulp of fresh mandarin oranges (*Citrus unshiu* Marc.), which were purchased from a local market in Seoul, South Korea. The normal (Madin-Darby Canine Kidney, MDCK) and cancer (Human cervical carcinoma, HeLa) cells (American Type Culture Collection; Manassas, VA, USA) were grown in Dulbecco’s modified Eagle’s medium containing 0.01% (*w/v*) penicillin and 10% (*v/v*) fetal bovine serum (FBS). The cultures were incubated with 5% CO_2_ at 37 °C. The culture medium, penicillin, and FBS were purchased from Gibco BRL (Grand Island, NY, USA).

### 2.2. Carotenoid Extraction and Saponification

The extraction and purification were conducted in the shortest possible time, to avoid degradation and isomerization [21]. In the first step, 1 kg of fruit pulp was separated and homogenized with 1 L hexane using a mechanical homogenizer (Daihan Scientific, Wonju-si, Korea). Homogenized samples were transferred to 200 mL centrifuge bottles, and centrifuged at 7000× *g* for 10 min at 4 °C, and the supernatant containing carotenoids was then recovered. The pelleted sample was repetitively (2–3 times) extracted using hexane, until they were colorless. The collected supernatants were pooled, partitioned, and the upper hexane phase was collected. The partitioning between upper hexane and the lower water phase was improved by adding ~10% (*v/v*) of 1 M sodium chloride. The hexane fraction was evaporated in a vacuum-rotary evaporator (Büchi, Switzerland) at 35 °C. The obtained extract was dissolved in 20 mL hexane, mixed with equal volume of 10% methanolic-potassium hydroxide (KOH; *w/v*), flushed with nitrogen (N_2_) gas (to minimize the oxidation), and incubated at 55 °C for 45 min for saponification. The mixture was transferred into a separating funnel, extracted thrice with hexane containing 10% diethyl ether, and the upper lipophilic hexane solutions were pooled, and then washed three times with water to remove traces of KOH. Diethyl ether was added to improve the polarity of hexane solution and enhance the solubility of non-esterified β-cryptoxanthin. The lipophilic hexane solution was dried under vacuum (<35 °C) using a rotary evaporator, and the residue was re-dissolved in 10 mL acetone.

### 2.3. Purification of β-Cryptoxanthin

The β-cryptoxanthin was isolated from the saponified sample according to the method described by Gansukh et al. [10]. The saponified sample (200 µL) was spotted to the baseline (above 1 cm) of 1500 μ silica gel preparative thin-layer chromatography (TLC) plates (Analtech, Newark, DE, USA) [10]. The TLC plates were chromatographed using a mixture of acetone/hexane (1:3; *v/v*). The major spot of β-cryptoxanthin (Rf = 0.6) was scraped, eluted with acetone, and centrifuged at 10,000× *g* for 5 min. The supernatant was collected, dried under nitrogen, and stored at −20 °C, until spectrophotometry, high-performance liquid chromatography (HPLC), atmospheric-pressure chemical ionization (APCI)-mass spectrometry (APCI-MS), APCI-tandem mass spectrometry (APCI-MS/MS or APCI-MS2) analysis, and the subsequent cell culture studies. 

### 2.4. Spectrophotometry, HPLC, APCI-MS, and APCI-MS/MS Analysis of β-Cryptoxanthin

For the quantification of β-cryptoxanthin, 1 mL of isolated β-cryptoxanthin was filtered through a Whatman (0.45 µm) filter, and the solution was then diluted with light petroleum. The absorbance (449 nm) was measured by UV-Visible spectrophotometry (Shimadzu, Japan, Model UV-2550). The β-cryptoxanthin concentration was determined using the molar absorption coefficient and absorbance values [22].

The percent purity of isolated β-cryptoxanthin in the filtered sample (acetone) was determined using HPLC (Agilent 1100, Agilent Technologies, Mississauga, ON, Canada) with a dual pump and diode array detector (DAD) set at 200–800 nm. The separation was achieved in a YMC C-30 carotenoid column (250 × 4.6 mm, 5 μm; YMC, Wilmington, NC, USA) at 20 °C. The solvent system was comprised of (A) methanol:water (95:5; *v/v*) containing 5 mM ammonium formate, and (B) tert-butyl methyl ether:ethanol (91:9; *v/v*), and the flow rate was 1 mL/min. The solvent elution was followed from (0 to 100)% B for 45 min. The injection volume was 20 µL. The β-cryptoxanthin was detected at 450 nm [23]. 

The APCI-MS and APCI-MS/MS analyses were performed by SCIEX API 3200 triple quadrupole mass spectrometer (AB-SCIEX, Redwood City, CA, USA) equipped with an Exion LC™ system and Turbo V™ interface with Heated Nebuliser™ (HN) probe. MS and MS/MS analysis of isolated β-cryptoxanthin were performed in APCI^+^ mode, following the liquid chromatographic (LC)-separation achieved using the above described HPLC conditions. The APCI^+^-MS and APCI^+^-MS/MS parameters were optimized as follows: Dry gas, N_2_; collision gas (CAD) 5 psi; curtain gas (CUR), 30 psi; ion source gas (GAS1), 45 psi; GAS2, 5 psi; temperature, 450 °C; nebulizer current, 4 nA; entrance potential (EP), 10; delustering potential (DP), 100 V; collision energy (CE), 60 V; collision cell exit potential (CXP), 10 V. Q1 MS and MS/MS mass spectra were acquired in the range of 100–600 *m/z* at 1 s interval.

### 2.5. Cytotoxic Activities of Purified β-Cryptoxanthin

The cytotoxicity of β-cryptoxanthin was assessed by a sulforhodamine B (SRB) assay [10,11]. HeLa and MDCK cells at a concentration of 1.5 × 10^5^ cells/mL were separately cultured in a 96-well plate, and incubated under 5% CO_2_ for 12 h at 37 °C. The growth medium was discarded, and the cells were washed carefully with 1× PBS (phosphate-buffered saline). The fresh growth medium containing 0.1, 1.0, 10, and 50 μM of β-cryptoxanthin was added to the wells containing HeLa and MDCK (in triplicates), and incubated for 24–48 h. The culture medium was discarded, washed carefully with 1× PBS, and then cells were fixed with 70% (*v/v*) of cold acetone (30 min, −4 °C), and finally dried in a hot air oven at 55–60 °C. Subsequently, 100 µL of SRB solution (0.4% *w/v* in 1.0% (*v/v*) acetic acid) was added to the wells, and the cells were incubated at 25 °C on a rocket shaker. After 24 h, the SRB solution in the wells was discarded, the cells were washed several times with 1% (*v/v*) acetic acid, and then completely dried in a hot air oven at 55–60 °C. The morphological changes were observed using a Zeiss Axiovert 200 M inverted microscope (Carl Zeiss, Oberkochen, Germany). The viability of cells was assessed by measuring the absorbance at 565 nm and percentage cell viability was calculated using the following formula;

$$Cell\;viability\;\left(\%\right)=100-\left(\frac{Ac-At}{Ac}\right)\times 100$$

*Ac* = absorbance of the control (untreated) cells, *At* = absorbance of cells treated with various concentrations of the β-cryptoxanthin.

### 2.6. RNA Isolation and Quantitative Real-Time PCR (qPCR) Analysis

The total RNA was extracted from HeLa cells using a TRIZOL reagent kit (Invitrogen, USA), using the manufacturer’s protocol. The quantification of isolated RNA was achieved using a NanoDrop 2000 spectrophotometer (Thermo Fisher Scientific, Middletown, VA, USA). The extracted RNA (2 μg) was used as a template to synthesize cDNA with the First Strand cDNA synthesis kit (Thermo Fisher Scientific, Middletown, VA, USA), according to the manufacturer’s instructions. Table S1 of the Supplementary Materials shows the sequences of primers used in the qPCR analysis of p53, Bax, Bcl-2, caspase-3, caspase-7, caspase-9, and glyceraldehyde-3-phosphate dehydrogenase (GAPDH) genes (Table S1). The qPCR analysis was carried out using the SYBR Green Master Mix (Bioneer, Oakland, CA, USA), according to the manufacturer’s instructions. The GAPDH gene is used to normalize the expression levels of the studied genes. The 2^−ΔΔCT^-based method was used to calculate the relative gene expression [24].

### 2.7. ROS Production Assay

ROS production was measured according to the method described previously [24]. The MDCK and HeLa cells were separately cultured at a concentration of 2 × 10^4^ cells/well in 6-well plates, and incubated under 5% CO_2_ at 37 °C. After 24 h, 0 or 250 µM of H_2_O_2_ was added to cells to stimulate the ROS production. Then β-cryptoxanthin at a concentration of 1.0 and 10 µM was added to both the ROS-stimulated and the control cells and maintained for 24 h. Cells were then incubated with 10 µM of 5-(and-6)-carboxy-2′,7′-dichlorofluorescein diacetate (Carboxy-H_2_DCFDA; Merck KGaA, Darmstadt, Germany) for 15 min at 37 °C, followed by three washes with PBS. Subsequently, the ROS level was assessed by a microplate spectrofluorometer (SpectraMax Gemini EM, Molecular Devices, Sunnyvale, CA, USA). Values are presented as relative fluorescent units (RFU).

### 2.8. Immunofluorescence Assay for Native Caspase-3 and the Integrity of the Mitochondrial Membrane 

HeLa cells at a density of 2 × 10^4^ cells/well seeded on a confocal culture dish were treated with 0, 1, and 10 μM of β-cryptoxanthin for 24 h. The cells were collected from the lower wells, rinsed with PBS, fixed with 4% (*v/v*) paraformaldehyde for 15 min, then permeabilized with 0.1% (*v/v*) Triton X-100 in PBS for 20 min, washed two times with ice-cold PBS, blocked in 3% (*w/v*) bovine serum albumin (BSA) for 40 min, and incubated with primary antibody (Asp175; Cell signaling technology, Danvers, USA) at 4 °C. After 3 h, HeLa cells were rinsed five times with PBS, before secondary antibody staining with fluorescein (FITC) donkey anti-rabbit IgG (H + L). The cells were finally washed two times with PBS [10]. Additionally, Mito-Red (Santa Cruz Biotechnology, South Korea) and Hoechst 33258 (Dojindo, South Korea) staining were performed to detect the integrity of the mitochondrial membrane and apoptotic nuclei, respectively. These confocal culture dishes were mounted with a fluorescent mounting media and analyzed with a Zeiss LSM-800 microscope (Carl Zeiss, Germany). Images were taken with the Zen-Black Edition software (Zen 2.3 SP1, Version: 14.0, Carl Zeiss, Germany).

### 2.9. Expression of Active (Cleaved) Caspase-3 Proteins

The cellular proteins were extracted and separated by sodium dodecyl sulfate (SDS)-polyacrylamide gel electrophoresis using 10% polyacrylamide, and then transferred to a polyvinylidene fluoride (PVDF) membrane (GVS Filter Technology, Stanford, ME, USA). To block nonspecific binding sites, membranes were maintained in 5% nonfat skim milk (Becton Difco, Sparks, MD, USA) for overnight at 4 °C on the shaker. Subsequently, membranes were incubated with the primary antibodies of caspase-3 (sc-7148; Santa Cruz Biotechnology, Santa Cruz, CA, USA), β-actin (sc-47778; Santa Cruz Biotechnology, Santa Cruz, CA, USA), and cleaved caspase-3 (#9661, Cell Signaling Technology, Danvers, MA, USA) for 3 h at room temperature (RT). Afterward, the membranes were incubated with secondary antibodies of horseradish peroxidase-conjugated mouse anti-rabbit (sc-2357; Santa Cruz Biotechnology, Santa Cruz, CA, USA) or anti-mouse conjugates (sc-516102; Santa Cruz Biotechnology, Santa Cruz, CA, USA). The membrane bands corresponding to the proteins were detected with enhanced chemiluminescence (SuperSignal West Femto Enhancer Kit; Pierce, Rockford, Loves Park, IL, USA). The membrane band images were obtained by iBright CL1000 Smart digital imager (Invitrogen, Carlsbad, CA, USA).

### 2.10. Terminal Deoxynucleotidyl Transferase-Mediated dUTP Nick End Labeling (TUNEL) Assay

The TUNEL assay was carried out using Click-iT TUNEL Alexa Fluor 647 kit (Thermo Fisher Scientific, Middletown, VA, USA), according to the manufacturer’s instructions. Briefly, HeLa cells at a concentration of 1.5 × 10^5^ cells/mL were cultured in glass-bottom confocal culture dishes, and incubated under 5% CO_2_ for 24 h at 37 °C. The cells were rinsed with PBS and treated with (0, 1.0, and 10) µM of β-cryptoxanthin for 24 h, and then incubated with 4% (*v/v*) formaldehyde for 20 min, followed by 0.25% (*v/v*) Triton X-100 for 25 min. TUNEL images were obtained by Olympus FLUOVIEW FV1200 microscope (Olympus Corporation, Japan). The TUNEL-positive cells were assessed by manual counting of at least 300 cells. Cells with only nuclear staining were considered TUNEL-positive (cytoplasmic staining was not considered).

### 2.11. Statistical Analyses

Each experiment was performed three times, and the values are expressed as mean ± standard deviation (SD). Data were subjected to ANOVA using SPSS Statistics 22 (IBM Inc., Armonk, NY, USA). Differences between treatments were calculated by Tukey’s test at *p* < 0.05.

## 3. Result and Discussion

### 3.1. Purification of β-Cryptoxanthin

**Figure 1:**
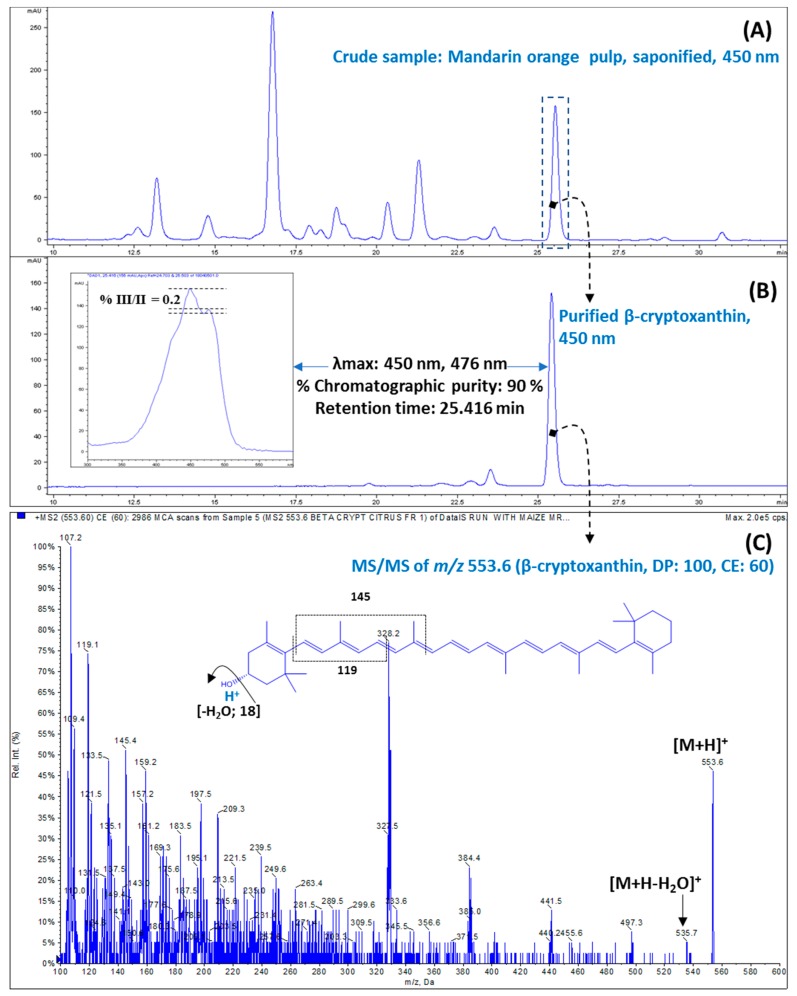
Isolation and characterization of β-cryptoxanthin. The high-performance liquid chromatography (HPLC) chromatogram of (**A**) a crude sample, and (**B**) isolated β-cryptoxanthin, showing λ_max_ and spectral fine structure. (**C**) The fragmentation pattern (tandem mass spectrometry (MS2) of *m/z* 553.6) of the β-cryptoxanthin molecule recorded using atmospheric-pressure chemical ionization (APCI)^+^ mode.

In this investigation, β-cryptoxanthin was successfully isolated from the pulp of fresh mandarin orange with 90.0% purity using a preparative TLC (Figure 1A,B). The β-cryptoxanthin was identified by the following parameters: (i) Retention time in HPLC (Figure 1B); (ii) absorption spectrum (λmax and spectral fine structure) (Figure 1B); and (iii) pattern of mass fragmentation (Figure 1C). Visible spectra (λmax of 450 and 476 nm) recorded during HPLC-DAD, spectral fine structures (denoted as %III/II) of 0.2, APCI^+^-Q1 mass of m/z 553.6, and MS2 of *m/z* 553.6 confirmed the identity of purified β-cryptoxanthin [25,26]. The characteristic positive ions at m/z 553.6 [M+H-H_2_O]^+^ due to the presence of hydroxyl group (−OH) on β-ione ring further validated the identity of isolated β-cryptoxanthin [27].

In the present investigation, a total of ~1500 μg of β-cryptoxanthin was purified from 20 preparative TLC plates, which were found adequate for performing the anticancer studies.

### 3.2. Proliferation Inhibitory Effect of β-Cryptoxanthin on HeLa Cells

**Figure 2:**
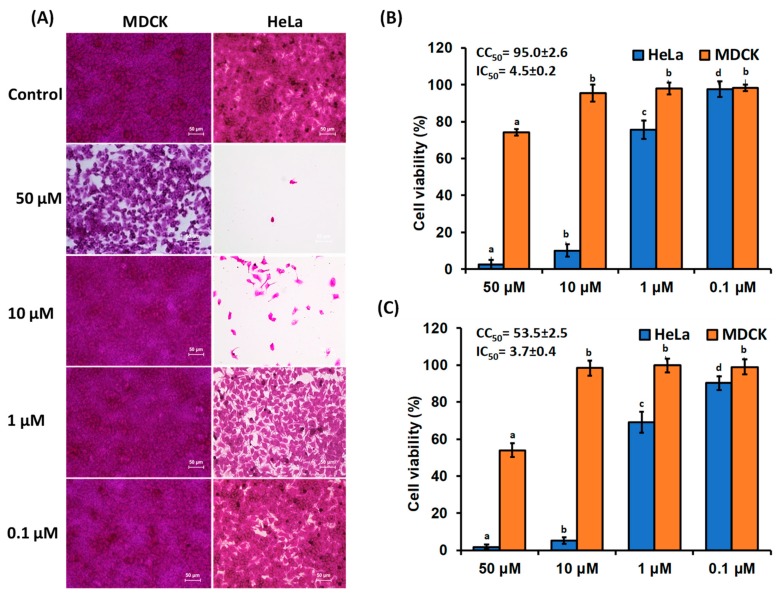
The effects of β-cryptoxanthin treatments on the proliferation of the normal Madin-Darby Canine Kidney (MDCK) and human cervical carcinoma (HeLa) cells. The dose of 0.1–50 μM of β-cryptoxanthin were used to treat MDCK and HeLa cells. (**A**) Analysis of morphological changes of normal MDCK and HeLa cells, documented after 48 h of incubation using light microscopy. The normal MDCK and HeLa cells viability after (**B**) 24 and (**C**) 48 h of incubation. The IC_50_ and CC_50_ indicate the 50% inhibition concentration of HeLa cell and 50% cytotoxic concentration of MDCK cells, respectively. All data are reported as mean values ± SD from independent experiments (*n* = 3). The means accompanied by different alphabets (e.g., a, b, c, and d) are significantly different at *p* < 0.05. Scale bar, 50 μm.

SRB assays were performed to determine the antiproliferation and cytotoxic effect of β-cryptoxanthin of 0.1–50 μM on HeLa cells and normal MDCK cells. The results showed that the β-cryptoxanthin treatment potentially inhibited the multiplication of HeLa cells in a concentration-dependent manner (Figure 2). Moreover, β-cryptoxanthin treatment did not affect the growth of normal MDCK cells. β-cryptoxanthin at a concentration of 10 μM reduced the multiplication of HeLa cells by up to 89.9% and 94.7% after a period of 24 and 48 h, respectively. The 50% inhibition concentration (IC_50_) of HeLa cells proliferation was recorded at 4.5 and 3.7 µM after 24 and 48 h of treatment, respectively (Figure 2). In this study, a very high concentration of β-cryptoxanthin (50 µM) was used only to evaluate the dose-dependent relationship for the inhibition of the multiplication of HeLa cells. However, this high concentration is not possible to achieve under the in vivo conditions. In a cohort study, 0.70–0.87 μM of β-cryptoxanthin was recorded in the serum from the Japanese population, which may substantially increase up to 4.12 µΜ after supplementation with β-cryptoxanthin-rich diet (e.g., Satsuma mandarin juice 125 mL/day for 12 weeks).

Earlier studies showed that the β-cryptoxanthin treatments of 5–20 μΜ can significantly reduce the multiplication of premalignant A549 and malignant lung cancer BEAS-2B cells [20], stomach tumor BGC-823 cells [18], and human colon adenocarcinoma Caco-2 cells [19]. Similarly, in a comparative study of the anticancer potential of 15 kinds of carotenoids, neoxanthin, fucoxanthin, phytofluene, ζ-carotene, and lycopene at 5–20 µM concentrations showed cytotoxicity against LNCaP, PC-3, DU 145 cells. However, β-cryptoxanthin and zeaxanthin did not affect the proliferation of these cells [28].

### 3.3. β-Cryptoxanthin Triggers ROS Production in HeLa Cells

ROS, including superoxide (^•^O^−^_2_), singlet oxygen (^1^O_2_), and lipid peroxyl radicals, play a vital role in the progress of chronic conditions and degenerative diseases, including CVD, neurodegenerative disorders, and cancer [29]. Cancer cells generate significantly higher levels of ROS than normal cells, due to their increased metabolic activities, oncogenic stimulation, and mutations in mitochondrial or nuclear genes, and dysfunctioning of mitochondrial electron transport chain [30]. However, further increase in ROS levels or decreased ROS scavenging capacity is cytotoxic to cancer cells, as it may push a cancer cell beyond the breakage point in terms of DNA damage, lipid peroxidation, and protein oxidation, which can lead to programmed cell death [31]. This approach of triggering ROS production is recently implicated by cancer chemotherapeutic agents, including carotenoids [32].

**Figure 3:**
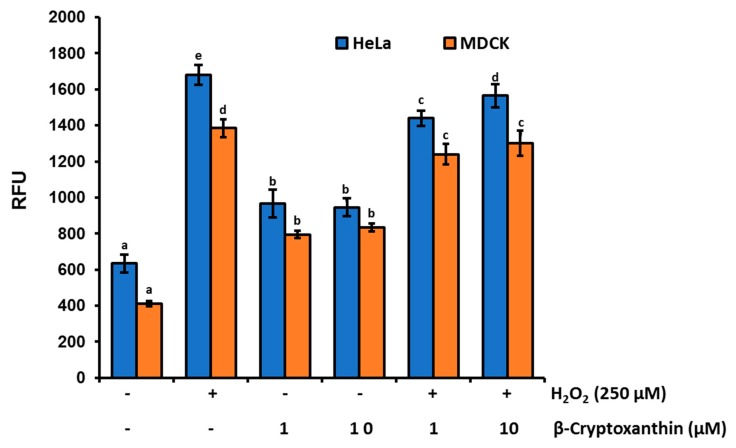
The effect of β-cryptoxanthin treatments on cellular ROS productions and accumulations. The enhanced levels of cellular ROS in both normal MDCK and HeLa cells were triggered by treatments with 250 µM H_2_O_2_, followed by treatment with β-cryptoxanthin (1.0 and 10 μM) for 24 h. Relative fluorescence of ROS was assessed by a microplate spectrofluorometer. All data are reported as mean values ± SD from independent experiments (*n* = 3). The means accompanied by different alphabets (e.g., a, b, c, d, and e) are significantly different at *p* < 0.05.

The enhanced levels of ROS generation have shown a crucial step in carotenoids-induced apoptosis of a variety of cancer cells [33], including fucoxanthin in human leukemia cell HL-60 cells [34], lycopene oxidation products in human prostate cancer MCF-7, PC-3, and HeLa cells [35,36], and lutein in HeLa cells [10]. Therefore, we analyzed whether β-cryptoxanthin could also trigger ROS production in Hela cells. After 24 h of incubation, cellular ROS levels evaluated by the cell-permeable fluorescent probe carboxy-H_2_DCFDA displayed significantly lower levels of ROS accumulation in normal MDCK cells (412 ± 15.6 RFU), compared to HeLa cells (635.0 ± 49.5 RFU) (Figure 3). When cells were treated with 10 μM β-cryptoxanthin, cellular ROS levels were increased significantly to 945.0 ± 49.5 RFU in HeLa cells, and 835.0 ± 21.2 RFU in MDCK cells. Similarly, ROS levels in both MDCK and HeLa cells were upregulated significantly by 250 µM H_2_O_2_ treatments (Figure 3). However, these increased levels of ROS in H_2_O_2_ treated cells were slightly attenuated by β-cryptoxanthin. It is fascinating to note that ROS levels are always lower in normal MDCK cells, compared to those in HeLa cells. Moreover, β-cryptoxanthin treatments decrease the ROS levels in highly oxidative stressed cells (e.g., H_2_O_2_ treated), while they induce ROS levels in normally proliferating HeLa cells. This dynamic control of ROS levels is probably modulated by both the pro-oxidant and antioxidant action of carotenoid [36,37,38].

At low oxygen pressure (pO_2_), carotenoid molecule can act as a powerful chain-breaking antioxidant. Meanwhile, at high pO_2_, they are readily autoxidized, and thus display pro-oxidant actions [38]. In the present investigation, probably under the physiological conditions of higher levels of basal ROS, decreased ROS scavenging capacity, and high oxygen tension of HeLa cells, β-cryptoxanthin acted as a pro-oxidant molecule, resulting in further elevation of ROS levels, thus triggering the oxidative stress-induced apoptosis of HeLa cells [10,36].

### 3.4. β-Cryptoxanthin Regulates the Expression of Apoptosis-Related mRNA, Proteins, and Disrupt the Integrity of the Mitochondrial Membrane

The rational activities of multidomain anti-apoptotic Bcl-2 and pro-apoptotic Bax proteins are crucial in maintaining the mitochondrial dynamics [39]. In the intrinsic pathway of apoptosis, intracellular stress, such as high level of ROS triggers enhanced the expression and translocation of pro-apoptotic Bax proteins from the cytosol to the outer mitochondrial membrane (OMM). This process facilitates the mitochondrial membrane permeabilization and dissipation of inner mitochondrial membrane potential (ΔΨm), resulting in matrix swelling, rupturing of the OMM, and leakage of cytochrome c and other apoptotic mediators into the cytosol [40]. Subsequently, these apoptotic mediators activate caspases, a large family of cysteine-aspartic protease executes programmed cell death [41]. The intrinsic pathway of mitochondria-mediated apoptosis is mainly modulated through the activation of caspase-9 (called initiator caspase). While, caspase-3 and -7 are the key downstream transducer (effector caspase; common to both intrinsic and extrinsic apoptotic pathways), and upon activation by an initiator caspase, it executes apoptosis by cleaving cellular substrates [42].

**Figure 4:**
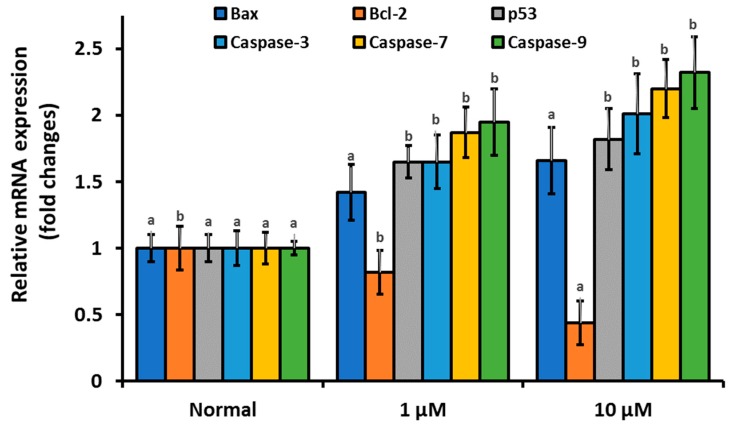
The mRNA expression profile of anti-apoptotic Bcl-2 and pro-apoptotic tumor protein p53, Bax, and caspase-3, -7, and -9 in HeLa cells treated with β-cryptoxanthin (1.0 and 10 μM) for 24 h. All data are reported as mean values ± SD from independent experiments (*n* = 3). The means accompanied by different alphabets (e.g., a and b) are significantly different at *p* < 0.05.

To confirm that the antiproliferation and cytotoxic activities of β-cryptoxanthin against HeLa cells are modulated by an intrinsic (mitochondrial) pathway of apoptosis, we utilized qPCR to determine the mRNA expression levels of the apoptosis-related gene in β-cryptoxanthin treated cells. The results revealed that mRNA expression levels of all pro-apoptotic genes, including caspase-3, -7, and -9, Bax, and p53 were upregulated significantly in HeLa cells treated with β-cryptoxanthin (1 and 10 μM for 24 h; Figure 4). In contrast, anti-apoptotic Bcl-2 mRNA expression was decreased significantly in HeLa cells treated with β-cryptoxanthin, in a dose-dependent manner.

**Figure 5:**
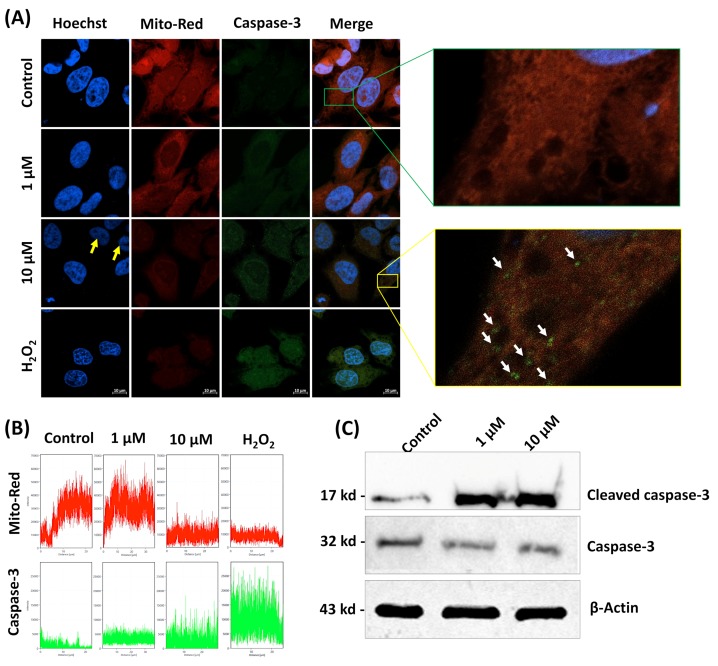
(**A**) Confocal observation of native caspase-3 protein expression (immunofluorescence assay), the integrity of the mitochondrial membrane, and Hoechst 33258 observations of apoptotic nuclei. In Hoechst 33258 stained HeLa cells, the yellow arrow indicates the apoptotic nuclei. Weak nuclear staining in β-cryptoxanthin (10 µM) treated cells probably caused by condensation and fragmentation of the DNA. The white arrows indicate the enhanced expression of native caspase-3 proteins. (**B**) The red and green fluorescent intensity show the integrity of the mitochondrial membrane and caspase-3 expression, respectively. The x-axis represents the horizontal distance (µm) across the cell, and the y-axis represents fluorescence intensity. (**C**) Cleaved caspase-3 protein expression by western blotting analysis. Scale bar, 10 μm.

The results of immunofluorescence assay also displayed the significantly enhanced expression of native caspase-3 protein in β-cryptoxanthin (1.0 and 10 μM) treated HeLa cells, compared to controls (Figure 5A,B). Additionally, western blot assay revealed the enhanced activation and cleavage of caspase-3 proteins in β-cryptoxanthin treated (1.0 and 10 μM) HeLa cells compared with controls (Figure 5C).

A decrease in cell viability, dephosphorylation mediated inhibition of bad expression, caspase-3 triggered poly-(ADP-ribose) polymerase cleavage, enhanced intracellular Ca^2+^ influx, and increased levels of ROS and RNS have been documented in human colon adenocarcinoma Caco-2 cells treated with β-cryptoxanthin and major dietary phytosterols, including β-sitosterol, campesterol, and stigmasterol [19]. Similarly, in our recent study, lutein-treated HeLa cells also showed enhanced accumulation of ROS correlated with significant downregulation of Bcl-2 and upregulation of Bax mRNA expression [9]. Upregulation of Bax and p53 and downregulation of Bcl-2 and cyclin D1 have also been observed in MCF-7 breast cancer cells treated with astaxanthin in combination with lutein and β-carotene [43].

In the present investigation, the apoptosis induction potential of β-cryptoxanthin was further confirmed by nuclei staining with Hoechst H33258. Apoptotic nuclei, as shown by chromatin condensation, were apparent after β-cryptoxanthin treatments for 24 h in a dose-dependent manner (Figure 5A). Weak nuclear staining probably caused by fragmentation and condensation of the DNA in β-cryptoxanthin (10 µM) treated cells [44]. The treatments with crocin have been shown to induce morphological changes, including chromatin condensation and DNA fragmentation in resistant (C13) and sensitive (OV2008) human cervical cancer cells after 48 and 72 h of treatments, respectively. Similarly, lycopene autoxidation products have displayed apoptotic nuclear changes in the HL-60 cells [45].

In further experiments, the confocal observation with Mito-Red dye on HeLa cells treated with β-cryptoxanthin (1.0 and 10 μM) revealed the loss of the integrity of the mitochondrial membrane and significant dissipation of intrinsic ΔΨm, evident from significant decrease in the fluorescence intensity of Mito-Red dye (Figure 5A,B). The evidence of β-cryptoxanthin triggered imbalance of Bcl-2 and Bax mRNA, significant disintegration of the mitochondrial membrane, and enhanced activation of caspase-3 proteins suggests the apoptosis of HeLa cells through the intrinsic pathway [40]. The treatments with β-carotene also showed apoptosis-inducing activities in human leukemia HL-60 cells, colon adenocarcinoma HT-29 cells, as well as melanoma SK-MEL-2 cell lines, via loss of ΔΨm, cytochrome c release, and activation of caspase-3 [46].

### 3.5. β-Cryptoxanthin Induces Nuclei DNA Fragmentation

**Figure 6:**
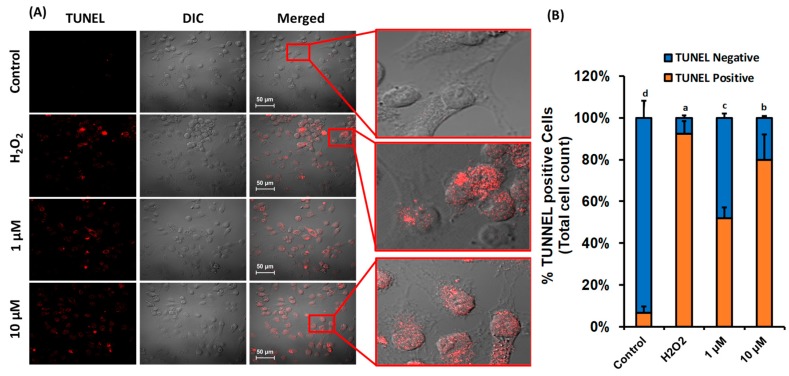
β-cryptoxanthin mediated HeLa cell nuclear DNA fragmentation detected by the TUNEL assay. (**A**) HeLa cells treated with β-cryptoxanthin (1.0 and 10 μM) for 24 h. The treatments with H_2_O_2_ were used as a positive control to induce nuclear DNA fragmentation. Scale bar, 50 μm. (**B**) The percentage of TUNEL positive and negative cell counts. The TUNEL-positive cells were quantified by manual counting of at least 300 cells. All data are reported as mean values ± SD from independent experiments (*n* = 3). The means accompanied by different alphabets (e.g., a, b, c, and d) are significantly different at *p* < 0.05. Scale bar, 50 μm.

Caspase triggered degradation of nuclear DNA is one of the hallmarks and the best characterized biochemical step of apoptotic cell death [47]. In the present investigation, the TUNEL assay revealed the significantly enhanced levels of nuclear DNA fragmentation in β-cryptoxanthin-treated HeLa cells. Only 6.7% of TUNEL-positive HeLa cells were recorded in 24 h control (untreated). Whereas, the proportions of TUNEL-positive cells were increased significantly in HeLa cells treated with 1.0 μM (52% TUNEL-positive cells) and 10 μM (80% TUNEL-positive cells) β-cryptoxanthin (Figure 6). Substantial damage to HeLa cells nuclei DNA in concordant with the enhanced activation of caspase-3 proteins suggested that the pro-apoptotic effects of β-cryptoxanthin are probably modulated by the activation of caspase-3 with consequent cleavage of nuclei DNA. Ganesan et al. [48] recorded 80% TUNEL-positive HL-60 human leukemia cells treated with 10 μM siphonaxanthin for 12 h. Likewise, fucoxanthin treatment has shown to induce nuclei DNA fragmentation in PC-3 human prostate cancer cells [49] and xenografted sarcoma 180 (S180) in male Kunming mice [50]. Neoxanthin and fucoxanthin also induce apoptosis of human prostate PC-3, DU 145, and LNCaP cell lines via nuclei DNA fragmentation [28].

To the best of the authors’ knowledge, no previous studies have described the β-cryptoxanthin-triggered apoptotic DNA fragmentation in HeLa cells. The 52% of TUNEL-positive cells recorded in the present investigation after β-cryptoxanthin treatment with the physiologically attainable concentration of 1.0 μM [51] which validates its potent bioactive properties. It might have potential as a chemopreventive drug of natural origin to minimize the risk of cervical carcinoma. Moreover, β-cryptoxanthin is a dietary provitamin A carotenoid; thus, the likelihood of adverse reactions of higher intake can be eliminated.

This study has several strengths. The β-cryptoxanthin used in the cytotoxicity studies was purified from a natural dietary source with high chromatographic purity of 90%. The expression of native as well as active caspase-3 proteins was analyzed by immunofluorescence and western blot assay. TUNEL assay validated the potential role of β-cryptoxanthin in cleavage of the HeLa cell nuclei DNA. On the other hand, our study has some limitations: (1) The intracellular ROS inhibitors were not tested, which may precisely justify the claim of ROS-triggered apoptosis of HeLa cells; (2) the use of mRNA data (except caspase-3) rather than measuring proteins by western blot; and (3) among active caspase proteins, the expression of only caspase-3 proteins was studied. The activities and expression of other proapoptotic (e.g., caspase-7 and -9, Bax, and p53) and antiapoptotic proteins (e.g., Bcl-2) can be studied in β-cryptoxanthin treated HeLa cells to further explore the cytotoxic mechanism.

## 4. Conclusions

In the present investigation, we provided substantial evidence of the β-cryptoxanthin-triggered enhanced accumulation of ROS in HeLa cells. Our results support our hypothesis that the β-cryptoxanthin-induced cytotoxicity against cervical cancer HeLa cells is linked to (1) enhanced ROS generation, (2) upregulation of caspase-3, -7, and -9, Bax, and p-53 at the mRNA level, with concordant downregulation of Bcl-2, (3) nuclear condensation and significant loss of the integrity of the mitochondrial membrane, (4) enhanced activation of caspase-3, and finally, (5) cleavage of nuclei DNA. The overall results indicate that β-cryptoxanthin could be used as a potential chemopreventive drug against human cervical carcinoma. In the future, the efficiency and possible synergism of β-cryptoxanthin with conventional chemotherapeutic drugs could be investigated for the effective treatment of human cervical carcinoma. Moreover, the activities of other key caspases, such as caspase-7 and -9 can be studied in β-cryptoxanthin treated HeLa cells to further explore the cytotoxic mechanism.

## Supplementary Materials

The following are available online at https://www.mdpi.com/2076-3921/9/1/28/s1. Table S1: qPCR primers list.
