# Supplementary material for: Chemopreventive Effect of β-Cryptoxanthin on Human Cervical Carcinoma (HeLa) Cells Is Modulated through Oxidative Stress-Induced Apoptosis
Source: Antioxidants (Basel). 2019 Dec 27;9(1):28. doi: 10.3390/antiox9010028 (PMC7022418; doi:10.3390/antiox9010028)
Supplement: Supplementary file 1 [file antioxidants-09-00028-s001.pdf]

Supplementary Information

qPCR primers list.

Type	Sequence	Bases
p53	5′-TAACAGTTCCTGCATGGGCGGC-3′	22
	5′-AGGACAGGCACAAACACGCACC-3′	22
Bax	5′-TGGAGCTGCAGAGGATGATTG-3′	21
	5′-GAAGTTGCCGTCAGAAAACATG-3′	22
Bcl-2	5′-CATGCTGGGGCCGTACAG-3	18
	5′-GAACCGGCACCTGCACAC-3′	18
Caspase-3	5′-TTAATAAAGGTATCCATGGAGAACACT-3′	28
	5′-TTAGTGATAAAAATAGAGTTCTTTTGTGAG-3′	30
Caspase-7	5′-GGAGAAAGCTCATGGCTGTGT -3′	21
	5′-TCCCCTTGGCTGTGTTTTG -3′	19
Caspase-9	5′-ATGACCACCACAAAGCAGTCC -3′	21
	5′-CGTGACCATTTTCTTGGCAG -3′	20
GAPDH	5′-CTTCACCACCATGGAGAAGGCTG-3′	23
	5′-GACCACAGTCCATGCCATCACTG-3′	23
